# Altered Topological Organization in the Sensorimotor Network After Application of Different Frequency rTMS

**DOI:** 10.3389/fnins.2019.01377

**Published:** 2019-12-19

**Authors:** Wei Wei, Tingting Zhu, Xiaoyu Wang, Lingyu Li, Qihong Zou, Yating Lv

**Affiliations:** ^1^Institutes of Psychological Sciences, Hangzhou Normal University, Hangzhou, China; ^2^Zhejiang Key Laboratory for Research in Assessment of Cognitive Impairments, Hangzhou, China; ^3^Center for MRI Research, Academy for Advanced Interdisciplinary Studies, Peking University, Beijing, China

**Keywords:** rTMS (repetitive transcranial magnetic stimulation), resting-state functional MRI, graph theory, sensorimotor network (SMN), primary motor cortex (M1)

## Abstract

The application of repetitive transcranial magnetic stimulation (rTMS) over the primary motor cortex (M1) could influence the intrinsic brain activity in the sensorimotor network (SMN). However, how rTMS modulates the topological organization of the SMN remains unclear. In this study, we employed resting-state fMRI to investigate the topological alterations in the functional SMN after application of different frequency rTMS over the left M1. To accomplish this, we collected MRI data from 45 healthy participants who were randomly divided into three groups based on rTMS frequency (HF, high-frequency 3 Hz; LF, low-frequency 1 Hz; and SHAM). Individual large-scale functional SMN was constructed by correlating the mean time series among 29 regions of interest (ROI) in the SMN and was fed into graph-based network analyses at multiple levels of global organization and nodal centrality. Our results showed that compared with the network metrics before rTMS stimulation, the left paracentral lobule (PCL) exhibited reduced nodal degree and betweenness centrality in the LF group after rTMS, while the right supplementary motor area (SMA) exhibited reduced nodal betweenness centrality in the HF group after rTMS. Moreover, rTMS-related alterations in nodal metrics might have been attributable to the changes in connectivity patterns and local activity of the affected nodes. These findings reflected the potential of using rTMS over M1 as an effective intervention to promote motor function rehabilitation.

## Introduction

Transcranial magnetic stimulation (TMS) is a focal and noninvasive technique that utilizes short, rapidly changing magnetic field pulses to induce electrical currents in underlying cortical tissue (Hallett, 2007; Fox et al., 2012). Repetitive TMS (rTMS) at different frequencies could induce distinct effects: high-frequency rTMS (>1 Hz) has been indicated to facilitate the cortical excitability of the ipsilateral hemisphere (Peinemann et al., 2004; Pascual-Leone et al., 2005; Khedr et al., 2009; Corti et al., 2012; Du et al., 2018), while low-frequency rTMS (≤1 Hz) could induce decreased cortical excitability in the ipsilateral side and increased excitability in the contralateral hemisphere (Muellbacher et al., 2000; Ziemann, 2004; Khedr et al., 2009; Corti et al., 2012; Du et al., 2018).

The application of rTMS over the primary motor cortex (M1), one of the vital brain areas responsible for motor control and execution, has been proven effective in several studies which explored its usage for promoting motor rehabilitation after stroke (Corti et al., 2012) or Parkinson’s disease (Lomarev et al., 2006; González-García et al., 2011). rTMS over M1 has not only induced brain activity changes in the stimulated area (Bestmann et al., 2003, 2004) but also influenced regions belonging to the sensorimotor network (SMN) which was spatially beyond the stimulated site (Bestmann et al., 2003, 2004; Yoo et al., 2008; Salinas et al., 2011, 2013, 2016). Bestmann et al. (2004) measured MRI signal changes during high-frequency rTMS (3.125 Hz) over the left primary sensorimotor cortex (M1/S1) with supra- and subthreshold intensity. They found high-frequency rTMS at different intensities activated a similar pattern of primary motor and sensorimotor regions such as supplementary motor area (SMA), premotor cortex, cingulate motor cortex and thalamus though subthreshold stimulation was with reduced effects. Yoo et al. (2008) examined the cortical activation following high-frequency rTMS (10 Hz) over right M1. The significant activations in the bilateral basal ganglia, left superior frontal gyrus, bilateral pre-SMA, right medial temporal lobe and right inferior parietal lobe associated with enhanced motor performance during a sequential finger motor task were detected. A baboon model developed by Salinas was employed to illustrate the cerebral blood flow (CBF) changes using the positron emission tomography (PET) imaging during suprathreshold rTMS over the left M1 at different high-frequency rates (3 Hz, 5 Hz, 10 Hz, 15 Hz). In addition to the stimulated M1 area, the significant increased CBF were found in regions within SMN (SMA and premotor cortex) for all high-frequency rTMS (Salinas et al., 2013). However, previous studies interested in the local activity alterations in regions within the SMN. The human brain has been revealed to integrate various inputs through multiple distributed systems and operate as a network (Barch, 2013; Sporns, 2014), which made the network approaches particularly suitable for the investigation of the human brain. Complex network analysis, one powerful tool to map the brain network, characterizes both intra- and inter-network connectivity patterns for the complete convergence of brain areas (Bassett and Bullmore, 2009; Craddock et al., 2013). Therefore, the complex network approaches could offer a more general view of the rTMS effects on the SMN.

Recent studies have applied multimodal neuroimaging data to construct the human brain networks with graph-based network analyses (Bullmore and Sporns, 2009; He and Evans, 2010). So far, several organizational principles of human brain networks have consistently been detected such as small-worldness (Liao et al., 2017) and the existence of hubs (van den Heuvel and Sporns, 2013). Resting-state functional MRI (rs-fMRI) measures spontaneous neuronal activity in human brain (Biswal et al., 1995) and has been commonly applied to explore the topological organization of brain functional networks (Wang et al., 2010). Park et al. (2014) demonstrated that increased global efficiency and decreased local efficiency of the whole brain network was observed in participants who had greater motor performance changes after employing high-frequency rTMS (10 Hz) over the right M1. However, how different frequency rTMS over M1 modulates topological organization in the SMN remains unclear.

In this study, we employed resting-state fMRI before and after the application of rTMS over left M1 to investigate the topological alterations in the SMN. Specifically, we sought to determine whether and how different frequency rTMS influences the topological organization of the SMN.

## Materials and Methods

### Participants

Forty-five right-handed healthy participants who had no history of neurological or psychiatric diseases and no contraindications for TMS and MRI were recruited from local universities (23 ± 2.67 years, 25 females) in the present study. The participants were randomly divided into three groups by rTMS frequency, including high-frequency group (HF, 3 Hz; *n* = 15, age = 24 ± 2.56 years, 8 females), low-frequency group (LF, 1 Hz; *n* = 15, age = 22.8 ± 3.1 years, 8 females) and sham group (SHAM; *n* = 15, age = 22.4 ± 2.16 years, 9 females). This study was approved by the Ethics Committee of the Center for Cognition and Brain Disorders at Hangzhou Normal University. All participants signed informed consents before attending the study.

### MRI Data Acquisition

In this study, we chose the offline measures which are intended to examine the plasticity induced by rTMS at different frequencies (Rounis et al., 2005; Yoo et al., 2008). Each participant underwent one MRI scan before and one scan after rTMS stimulation using the same imaging protocol. The second MRI scan was performed within 30 min after stimulation (HF, 13.93 ± 4.3 min; LF, 14.67 ± 5.01 min; SHAM, 12.4 ± 5.18 min).

Two MRI scans were performed on a GE 3T scanner (MR-750, GE Medical Systems, Milwaukee, WI) at the Affiliated Hospital of Hangzhou Normal University. Each MRI scan included the following three sessions:

*Resting-state fMRI*: Echo-planar imaging sequence, 43 axial slices, 240 volumes, repetition time (TR) = 2000 ms, echo time (TE) = 30 ms, field of view (FOV) = 220 × 220 mm^2^, voxel size = 3.44 mm × 3.44 mm × 3.20 mm, flip angle = 60°.

*Structural MRI*: 3D-MPRAGE sequence, 176 sagittal slices, TR = 8100 ms, TE = 3.1 ms, FOV = 256 × 256 mm^2^, voxel size = 1 mm × 1 mm × 1 mm.

*Task-fMRI*: acquired using the same parameters as the rs-fMRI. A block-designed finger-tapping task was performed in this session, which consisted of eight 20s task blocks and seven 20s rest blocks. The participants were instructed to press a key with right index finger following presentation of a red circle flashed at a frequency of 1 Hz during the task blocks, and to stare at a white cross in the center of screen during the rest blocks. This session was acquired after resting-state fMRI scanning session to exclude the effects of finger moves on the resting-state BOLD signal.

### rTMS Intervention

The rTMS was applied using a Magstim TMS machine (Magstim Inc., Sheffield, United Kingdom) equipped with a figure-of-eight coil. All applications of rTMS followed the safety guidance of rTMS provided by the International Workshop (Wassermann, 1998).

#### Resting Motor Threshold

Participants were instructed to sit and relax comfortably in an adjustable armchair. Motor evoked potential (MEP) amplitudes were recorded from abductor pollicis brevis (APB) muscle of right hand. For each participant, the target coordinates of left M1 were located in the hand knob area on structural image and were marked with Brainsight software^1^. The frameless stereotaxy was then applied to coregister the structural image to the head for each participant (Paus et al., 1997). Each participant’s head position was assessed using the Polaris infrared tracking system (Northern Digital, Waterloo, Canada) base on four landmarks (nasion, nose tip and intertragal notch of both ears) on the structural image. Single-pulse TMS was first delivered to target position while the coil was systematically moved in 1-cm increments at a constant suprathreshold stimulus intensity to detect the “hot spot” (Yoo et al., 2008), where the MEPs in the APB muscle could be evoked with maximum peak-to-peak amplitude and shortest latencies (Cárdenas-Morales et al., 2013). The resting motor threshold (RMT) was defined as the lowest stimulus intensity eliciting MEP amplitudes greater than 50 μV at least five times in 10 consecutive trials over the hot spot (Rossini et al., 1994; Rothwell et al., 1999). During the rTMS application, the surface electromyography (EMG) of the APB muscle was constantly recorded.

#### Location of TMS Target Region

Individual activation map from the right finger-tapping task was generated using SPM12^2^. For each participant, the activation map was then projected to the anatomical image using Brainsight software^1^. The most significantly activated voxel in the left anterior wall of the central sulcus was located as the individual TMS target for rTMS stimulation.

#### TMS Protocol

The coil was placed tangentially over the target region in left M1 after the anatomical coregistration using frameless stereotaxy. All stimulations were administered with the magnitude of the pulse set at 90% RMT.

*HF group*: High-frequency rTMS included five consecutive pulse blocks interleaved with 15 s of quitting time. Each block was composed of 300 pulses at a frequency of 3 Hz and lasted for 100 s. Each participant received a total of 1500 pulses over the course of 9.3 min. The motor threshold of HF group was 62 ± 6.5%.

*LF group*: Low-frequency rTMS also included five consecutive pulse blocks interleaved with 15 s of quitting time. Each block comprised 300 pulses at a frequency of 1 Hz and lasted for 300 s. Each participant received a total of 1500 pulses lasting 26 min. The motor threshold of LF group was 63 ± 6.3%.

*SHAM group*: For sham group, the coil was placed at a 90° angle to the skull and the stimulation parameters were same as the LF group.

### Resting-State fMRI Data Preprocessing

The resting-state fMRI data was processed with the GRETNA package (Wang et al., 2015), including the following steps: (1) discarding the first five volumes for signal equilibrium and participants’ adaptation to the scanning noise; (2) slice timing correction for the time delay between slices; (3) intervolume head motion correction; (4) coregistration of individual T1 images to the functional images; (5) spatial normalization to the Montreal Neurological Institute (MNI) space via deformation fields from tissue segmentation of the T1 images; (6) removing the linear trend of the time courses; (7) bandpass filtration (0.01–0.08 Hz); and (8) regressing out the head motion effect (Friston 24 parameter) (Friston et al., 1996), white matter and cerebrospinal fluid signals. Note that, all nuisance signals were also bandpass filtered (0.01–0.08 Hz) (Hallquist et al., 2013).

### Network Construction

**FIGURE 1:**
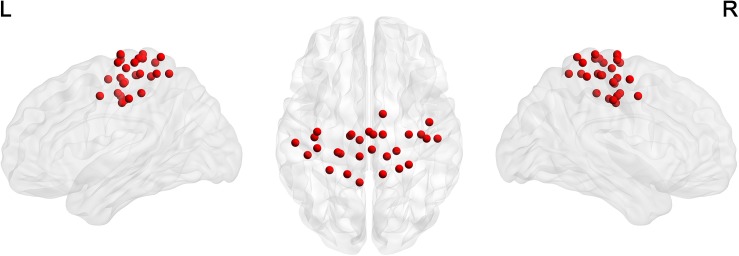
The distribution of 29 ROIs in the sensorimotor network (Power et al., 2011) was visualized in surface space using the BrainNet Viewer (Xia et al., 2013).

For the node definition, we extracted 29 non-overlapping sensorimotor regions of interest (ROI) from a functional brain atlas (Power et al., 2011) as nodes (Table 1 and Figure 1). We calculated the average of weighted blood oxygen level-dependent signals of all voxels in each ROI (with the weights representing gray matter probabilities) as the time course of that ROI. The Pearson correlation coefficients between time courses of each pair of ROIs were then calculated. Thus, we obtained a 29 × 29 correlation matrix for each participant.

**TABLE 1:** Regions of interest in the sensorimotor network.

**ROI**	**MNI coordinate**			**Regions**	**Side**	**Brodmann area**
	**X**	**Y**	**Z**			
1	−7	−52	61	Precuneus	L	BA5
2	−14	−18	40		L	
3	0	−15	47	Middle cingulum	L	
4	10	−2	45	Middle cingulum	R	
5	−7	−21	65	Paracentral lobule	L	BA4
6	−7	−33	72	Paracentral lobule	L	BA4
7	13	−33	75	Postcentral gyrus	R	BA4
8	−54	−23	43	Supramarginal gyrus	L	BA3
9	29	−17	71	Precentral gyrus	R	BA6
10	10	−46	73	Precuneus	R	BA5
11	−23	−30	72	Postcentral gyrus	L	BA4
12	−40	−19	54	Postcentral gyrus	L	BA4
13	29	−39	59	Postcentral gyrus	R	BA2
14	50	−20	42	Postcentral gyrus	R	BA3
15	−38	−27	69	Postcentral gyrus	L	BA4
16	20	−29	60	Precentral gyrus	R	BA3
17	44	−8	57	Precentral gyrus	R	BA6
18	−29	−43	61	Postcentral gyrus	L	
19	10	−17	74	Supplementary motor area	R	BA6
20	22	−42	69	Postcentral gyrus	R	
21	−45	−32	47	Postcentral gyrus	L	BA2
22	−21	−31	61	Postcentral gyrus	L	BA3
23	−13	−17	75	Paracentral lobule	L	BA6
24	42	−20	55	Postcentral gyrus	R	BA4
25	−38	−15	69	Precentral gyrus	L	BA6
26	−16	−46	73	Parietal superior	L	BA5
27	2	−28	60	Paracentral lobule	R	BA4
28	3	−17	58	Supplementary motor area	R	BA6
29	38	−17	45	Precentral gyrus	R	BA4

*ROI = regions of interest, MNI = Montreal Neurological Institute, L = left, R = right, BA = Brodmann area.*

To denoise spurious interregional connectivity, a sparsity-based thresholding procedure was employed to ensure the same network density for all participants. We repeatedly converted each matrix into a binary matrix by thresholding all correlation metrics in a sparsity range (from 0.04 to 0.4 at an interval as 0.02), which ensured that the resultant networks had sparse properties (Achard et al., 2006; Wang et al., 2009). It should be noted that the negative correlations were excluded due to the ambiguous interpretation and unfavorable influences on test–retest reliability (Fox et al., 2009; Murphy et al., 2009; Weissenbacher et al., 2009; Wang et al., 2011). We then performed the following network analyses at each sparsity threshold, which resulted in curves of sparsity for each of network metrics listed below.

### Network Analysis

After SMN construction, two global metrics (global efficiency and local efficiency) and three nodal centrality metrics (efficiency, degree, and betweenness) were further calculated for each network matrix to characterize their topological organization as previous study (Lv et al., 2019). These metrics were explained below in a binary network *G* with *N* nodes and *K* edges.

#### Global Metrics

Efficiency is an index which describe the network from the perspective of parallel information flow (Latora and Marchiori, 2001; Achard and Bullmore, 2007). The global efficiency is calculated with following equations:

(1) $$E_{g\,l\,o\,b}\,\left(G\right)=\frac{N}{N\,\left(N-1\right)}\,\sum_{i\neq j\in G}\frac{1}{d_{i\,j}}$$

The *d*_*ij*_, computed as the smallest sum of the edges throughout all the possible paths connects node *i* and node *j*, denotes the shortest path length between two nodes *i* and *j*. The global efficiency reflects the ability of parallel information transmission within the network.

The local efficiency of the network is the average of all nodal efficiencies, which is calculated as follows:

(2) $$E_{l\,o\,c}\,\left(G\right)=\frac{1}{N}\,\sum_{i\in G}E_{g\,l\,o\,b}\,\left(G_{i}\right)$$

The *E*_*glob*_(*G*_*i*_) is the global efficiency of the subgraph of node *i* (*G*_*i*_), which is comprised of nodes directly linking to node *i*. The local efficiency represents the capability of information exchange over each subgraph when the index node is removed.

The normalized local efficiency (${\tilde{E}}_{l\,o\,c}$) and normalized global efficiency (${\tilde{E}}_{g\,l\,o\,b}$) were further calculated by dividing each by the corresponding mean derived from 100 random networks with the same number of nodes, edges and degree distribution as the real network (Maslov and Sneppen, 2002; Milo et al., 2002). The network was topologically organized as a small-world if its normalized global efficiency was approximately equal to 1 and its normalized local efficiency was larger than 1 (Watts and Strogatz, 1998).

#### Nodal Centrality Metrics

For node *i* in a network, the nodal degree centrality is calculated as the sum of edges between node *i* and other nodes:

(3) $$N_{d\,c}\,\left(i\right)=\sum_{j\neq i\in G}a_{i\,j}$$

The nodal efficiency is computed as the reciprocal of the shortest path length between node *i* and all the other nodes in the network (Achard and Bullmore, 2007):

(4) $$E_{n\,o\,d\,a\,l}\,\left(i\right)=\frac{1}{N-1}\,\sum_{j\neq i\in G}\frac{1}{d_{i\,j}}$$

The nodal betweenness centrality is defined as follows:

(5) $$N_{b\,c}\,\left(i\right)=\sum_{j\neq i\neq k\in G}\frac{\sigma_{j\,k}\,\left(i\right)}{\sigma_{j\,k}}$$

where σ_*jk*_ represents the number of the shortest paths between node *j* and node *k*. σ_*jk*_(*i*) is the number of the shortest paths between node *j* and node *k* pass through node *i*. The area under the curve (the integral over the sparsity range), which is used for subsequent statistical analyses, were further calculated for each network metric of each participant.

### Statistical Analysis

The group difference in age and time intervals between rTMS and subsequent MRI scan were measured by one-way analysis of variance (ANOVA). The between-group difference of sex ratio was measured using the chi-square test. For two global network metrics (local efficiency and global efficiency) and three nodal centrality metrics (degree, efficiency, and betweenness), two-way repeated measures ANOVA was performed for each metric with three levels (HF, LF, and SHAM groups) as the between-subject factor and two levels (before and after TMS) as the within-subject factor by using SPSS (Statistical Product and Service Solutions, IBM, United States) software. For each network metric, Bonferroni corrections (*p* < 0.05) were applied for multiple comparisons. *Post hoc* comparisons were subsequently performed in those global and nodal metrics with significant interactions (stimulation frequency × MR scanning session) to compare rTMS effects in each group and group differences before or after rTMS application.

For any node showing significant rTMS-related alterations in nodal metrics affected by the rTMS, we subsequently examined functional connectivity (FC) patterns and local activity. For FC, the Pearson’s correlation coefficients between the average time courses of each node and the other 28 ROIs within the SMN were calculated and converted to *z*-values by Fisher’s *r*-to-*z* transformation. To characterize the local activity, the amplitude of low-frequency fluctuation (ALFF) of each node was calculated (Zang et al., 2007). The differences in FC and ALFF values of each node before and after rTMS application were inferred using paired *t*-tests. A Pearson correlation analysis was also performed to assess the associations between differences in FC or ALFF values and differences in nodal metrics of each node before and after rTMS.

## Results

### Demographic Characteristics

No adverse effects of TMS was reported by any of participants. No significant differences were found in age (HF, 24 ± 2.56 years; LF, 22.8 ± 3.10 years; SHAM, 22.4 ± 2.16 years; *F*_(2,12)_ = 1.496, *p* = 0.236), sex ratio (HF, 7 females; LF, 7 females; SHAM, 6 females; χ^2^ = 0.18, *p* = 0.914), and time intervals (between rTMS and the subsequent MRI scan) (HF, 13.93 ± 4.30 min; LF, 14.67 ± 5.01 min; SHAM, 12.40 ± 5.18 min; *F*_(2,12)_ = 0.855, *p* = 0.433) among the three groups.

### Global Organization of the Functional Sensorimotor Network

**FIGURE 2:**
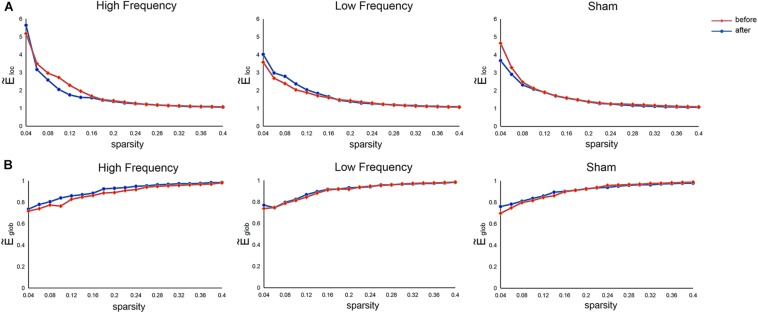
Mean normalized local efficiency **(A)** and mean normalized global efficiency **(B)** of the sensorimotor network before and after rTMS. ${\tilde{E}}_{l\,o\,c}$, normalized local efficiency; ${\tilde{E}}_{g\,l\,o\,b}$, normalized global efficiency.

Relative to the matched random networks, the SMN showed small-world organization with normalized local efficiency (${\tilde{E}}_{l\,o\,c}$) > 1 (HF = 1.78 ± 1.08; LF = 1.65 ± 0.73; SHAM = 1.65 ± 0.81) and normalized global efficiency (${\tilde{E}}_{g\,l\,o\,b}$) ≈ 1 (HF = 0.90 ± 0.08; LF = 0.91 ± 0.08; SHAM = 0.91 ± 0.08) (Figure 2). These findings suggested that the SMN had a high-efficiency network organization with small-world architectures. Nevertheless, no significant main effects of stimulation frequency (HF, LF, SHAM) and MR scanning session (before and after rTMS) and their interaction were observed in these global metrics.

### Local Nodal Characteristics of the Functional Sensorimotor Network

Repeated measures ANOVA showed no main effects of stimulation frequency and MR scanning session in nodal centrality metrics (betweenness centrality, degree centrality, and nodal efficiency), while significant interactions were observed in the left paracentral lobule (PCL) (*F*_(2,42)_ = 3.501, *p* = 0.039) and right SMA (*F*_(2,42)_ = 3.756, *p* = 0.032) for betweenness centrality and in the left PCL (*F*_(2,42)_ = 3.546, *p* = 0.038) for degree centrality.

**FIGURE 3:**
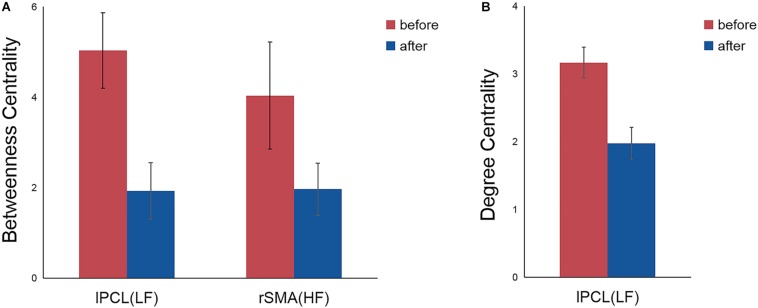
Significant differences in nodal betweenness centrality **(A)** and nodal degree centrality **(B)** before and after rTMS. Error bars indicate standard errors. lPCL, left paracentral lobule; rSMA, right supplementary motor area; LF, low-frequency group; HF, high-frequency group.

*Post hoc* comparisons subsequently showed that, compared with the nodal centrality measures before stimulation, stimulation decreased betweenness centrality (*p* = 0.007, Bonferroni corrected) and degree centrality (*p* = 0.001, Bonferroni corrected) in the left PCL in the LF group, while the right SMA exhibited decreased betweenness centrality in the HF group (*p* = 0.043, Bonferroni corrected) (Table 2 and Figure 3).

**TABLE 2:** The significant interaction effect in nodal topological metrics.

**Metrics**	**Region**	***F***	***P***	***Post hoc***	***P***
Betweenness centrality	L PCL	3.501	0.039	LF before > after	0.007
Betweenness centrality	R SMA	3.756	0.032	HF before > after	0.043
Degree centrality	L PCL	3.546	0.038	LF before > after	0.001

*L = left, R = right, PCL = paracentral lobule, SMA = supplementary motor area, LF = low frequency, HF = high frequency.*

**FIGURE 4:**
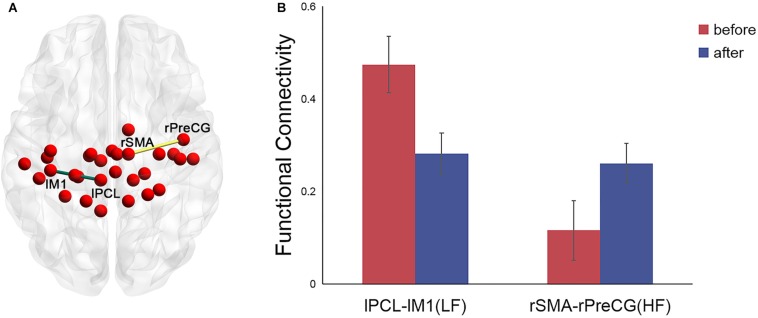
Significant differences in the functional connectivity (FC) patterns in the left PCL and right SMA in the functional sensorimotor network before and after rTMS. **(A)** Nodes with significantly changed FC were visualized in surface space; **(B)** FC between the left PCL and left M1 in the LF group and FC between the right SMA and right precentral gyrus in the HF group. Error bars indicate standard errors. lPCL, left paracentral lobule; lM1, left primary motor cortex; rSMA, right supplementary motor area; rPreCG, right precentral gyrus; LF, low-frequency group; HF, high-frequency group.

**FIGURE 5:**
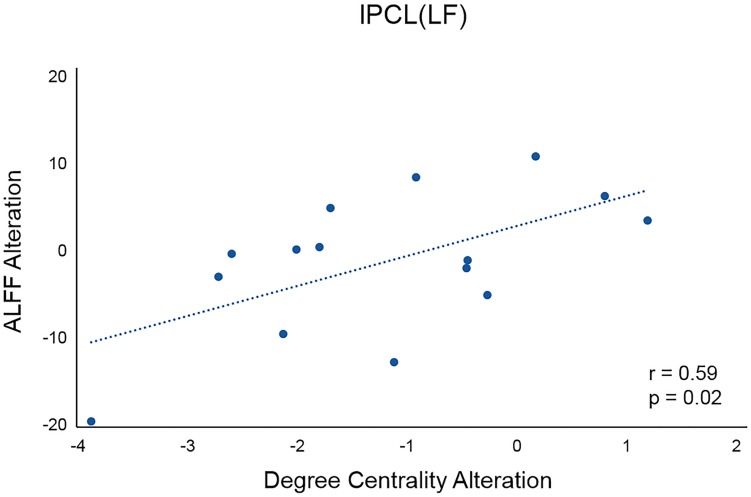
Correlation between the differences in ALFF and the differences in degree centrality in the left PCL before and after rTMS in the LF group. lPCL, left paracentral lobule; LF, low-frequency group.

For the left PCL and the right SMA, we further investigated the alterations of their FC patterns and ALFF values before and after application of different frequency rTMS. We found significantly decreased FC between the left PCL and left M1 (*p* = 0.006) in the LF group and significantly increased FC between the right SMA and right precentral gyrus (PreCG) (*p* = 0.004) in the HF group (Figure 4). However, there was no significant correlation between FC alterations and nodal centrality reductions in the left PCL and right SMA. No significant differences in ALFF of the left PCL and right SMA were observed before and after rTMS, while the differences in ALFF positively correlated with the differences in degree centrality in the left PCL before and after rTMS in the LF group (*r* = 0.5926, *p* = 0.0199) (Figure 5). That is, the more the ALFF values in the left PCL decreased, the more the degree centrality in the left PCL decreased.

## Discussion

In this study, we employed resting-state fMRI to investigate the topological alterations in functional SMN after application of different frequencies of rTMS over M1. We found that, compared with the network metrics before stimulation, the left PCL showed decreased nodal degree and betweenness centrality when applying low-frequency rTMS over the left M1, while the right SMA exhibited reduced nodal betweenness centrality after stimulation with high-frequency rTMS. Moreover, rTMS-related alterations in nodal metrics might have been attributable to the changes in connectivity patterns and local activity of the affected nodes. Overall, these findings provide evidence that rTMS may affect the topological organizations of functional SMN, which may help to elucidate the mechanisms justifying the application of rTMS in the treatment of patients with motor dysfunctions.

The human brain is a complex, interconnected network that continuously integrates information across distributed brain regions (Wang et al., 2013). Previous studies have demonstrated that the human brain networks exhibited several topological configurations, such as small-worldness, an efficient model to support within- and between-module information transfer (Bassett and Bullmore, 2017; Liao et al., 2017). Here, we observed small-world organizations of functional SMN in all participants before and after application of rTMS over the left M1, indicating an optimal balance between global integration and local specialization within the SMN. However, quantitative comparisons of network efficiency revealed no significant differences among the three groups before and after stimulation, which may suggest the preservation of an optimal wiring layout in the SMN after application of different frequency rTMS. Notably, no significant effect of rTMS on network efficiency may be a consequence of short stimulation time; that is, there was only one rTMS session for each participant. Future studies are needed to further explore whether multiple stimulation sessions may induce alterations in network efficiency.

At a nodal level, the three nodal centrality metrics quantifies the importance of a node in a network. Nodes with high centrality can be categorized as network hubs (Sporns et al., 2007; Buckner et al., 2009). In this study, rTMS-related decreases in centrality were mainly observed in two regions, the left PCL and right SMA, which may indicate that these two regions had dropped influence on the flow of information in the SMN.

In our study, compared to the nodal metrics before stimulation, decreased nodal degree and betweenness centrality in the left PCL, ipsilateral to the stimulation side, were observed after low-frequency rTMS. Specifically, decreased degree centrality was positively associated with decreased ALFF in the left PCL. These findings were consistent with the inhibitory effect of low-frequency rTMS on ipsilateral cortical excitability (Khedr et al., 2009; Corti et al., 2012; Du et al., 2018). The PCL, a U-shaped convolution on the medial hemispheric surface, connects medial portions of the precentral and postcentral gyrus and is involved in motor control and sensory innervations of the limbs (Johns, 2014). Therefore, we suspected that the reduced nodal degree and betweenness centrality in the left PCL may be relevant to reduced abilities in motor control and sensation, which is supported by decreased FC between the left PCL and left M1 after stimulation in the LF group. Low-frequency rTMS has been commonly applied over contralesional M1 in the treatment of stroke patients with motor dysfunction to suppress the excitability of unaffected hemisphere (Khedr et al., 2009; Corti et al., 2012; Du et al., 2018). The results suggested that the PCL may be a key structure for low-frequency rTMS to suppress the activity in the unaffected hemisphere. Thus, it is important to clarify the roles played by the PCL when using low-frequency rTMS for the treatment of stroke patients.

High-frequency rTMS has been shown to increase cortical excitability in the ipsilateral hemisphere (Peinemann et al., 2004; Pascual-Leone et al., 2005; Khedr et al., 2009; Corti et al., 2012; Du et al., 2018). However, in our study, the right SMA, contralateral to the stimulation site, showed decreased betweenness centrality after high-frequency rTMS. Thus, we propose that the high-frequency rTMS may inhibit the activity in the contralateral hemisphere. The SMA locates in front of PCL (Johns, 2014) and projects to both ipsilateral and contralateral and primary motor cortices (Pandya and Vignolo, 1971; Muakkassa and Strick, 1979). Recent fMRI studies have shown that the interhemispheric connections of the SMA played an important role in coordinating bimanual movements (Stanák et al., 2003; Seitz et al., 2004), especially in mediating intended actions and suppressing unintended movements. The SMA has a suppressive influence over contralateral M1 to prevent motor execution (Grefkes et al., 2008; Kasess et al., 2008). Previous studies in one patient with restricted lesion within the SMA also showed the automatic suppression of motor plans by the SMA (Nachev et al., 2007; Sumner et al., 2007). The results in our study implied that the decreased centrality in the right SMA may have reduced the inhibitory effect on the left M1, which means increased excitability on the stimulated side. Notably, no significant differences in FC between right SMA and left M1 before and after rTMS were observed, and thus, the reduced suppressive effect of the right SMA on the stimulated side after application of high-frequency rTMS needs to be elucidated in future studies.

Repetitive TMS is a non-invasive technique that could induce sustained influence on brain plasticity (Rounis et al., 2005; Yoo et al., 2008). However, the effect of rTMS depends on various factors, such as the frequency, intensity of stimulation, and the number of delivered stimuli. Generally, high-frequency rTMS tend to induce excitation of the motor network, while low-frequency rTMS can produce the inhibitory effect on cortical excitability. 1 Hz was the most commonly used when applying low-frequency rTMS (Corti et al., 2012; Hsu et al., 2012). Regarding the high-frequency rTMS, different frequency rates has been adopted in previous studies, such as 3 Hz, 5 Hz, 10 Hz (Chang et al., 2010; Emara et al., 2010; Khedr et al., 2010). However, it has been indicated that rTMS with higher-frequency rate was associated with increased electromyographic bursting and spread of excitation, which means higher risk of seizure (Lomarev et al., 2007). Moreover, Khedr et al. (2010) compared the long-term effect of 3 and 10 Hz rTMS on recovery of motor function in stroke patients and found that rTMS at 3 Hz seemed to produce greater changes in strength and clinical rating scales although not reached significant level. In this case, 3 Hz which adopted in our study may be a safer choice for high frequency stimulation, especially for the patients with motor dysfunctions. As the preliminary study for applying rTMS in the treatment of stroke patients, our study defined high-frequency as >1 Hz and low-frequency as ≤1 Hz which was also consistent with previous systematic review (Corti et al., 2012) and meta-analysis (Hsu et al., 2012). However, given that high-frequency rTMS at different frequency rates may induce different degree of impacts on brain’s plasticity (Khedr et al., 2010), it is important for future studies to examine the similarities and differences in their effects among these frequency rates. The intensity of rTMS has also been suggested as a critical factor of its effects. Previous studies demonstrated that suprathreshold (above RMT) rTMS at low frequency (1 Hz) could induce lasting inhibitory effect on cortical excitability (Chen et al., 1997; Muellbacher et al., 2000), and subthreshold (below RMT) stimulation at low frequency (1 Hz) could also decrease cortical excitability though with weaker aftereffects when compared with suprathreshold stimulation (Siebner et al., 1999; Touge et al., 2001; Sommer et al., 2002). Suprathreshold high-frequency rTMS tend to increase the corticospinal excitability (Pascual-Leone et al., 1994; Wu et al., 2000), while high-frequency rTMS with subthreshold intensity has been shown to induce different effects on cortical excitability: facilitatory (Maeda et al., 2000a, b) or inhibitory effects (Peinemann et al., 2000; Di Lazzaro et al., 2002; Todd et al., 2006). One possible explanation for this discrepancy is short period of stimulation (in other words, the small number of stimuli delivered), which has been proven to be an important factor of rTMS effects. Prolonged period (≥900 stimuli) of high-frequency rTMS at subthreshold intensities could increase overall cortical excitability (Touge et al., 2001; Quartarone et al., 2005). For example, Quartarone et al. (2005) showed 5 Hz rTMS with 90% RMT and 1500 stimuli provoked an overall increase in corticospinal excitability. Accordingly, 1500 stimuli delivered with subthreshold intensity (90% RMT) at high frequency in our study should induce the similar faciliatory effect on motor cortical excitability. However, our study only explored the effect of rTMS with subthreshold intensity, which is unable to reveal the alterations induced by rTMS with suprathreshold intensity. Future studies applying rTMS with both suprathreshold and subthreshold at different frequencies may help clarify their influence on the topological organizations of SMN.

There are several limitations in our study that need to be addressed. First, the stimulation time of rTMS may have been too short to detect topological alterations in the SMN. As the application of rTMS in the treatment of patients always lasts several weeks, future studies with longer stimulation time are required to examine the effects of rTMS on SMN. Second, we failed to collect behavioral data in this study, and thus cannot examine behavioral alterations in motor function. Future studies with behavioral data collected before and after rTMS can help clarify this issue. Finally, recent evidence indicated that increasing the acquisition time (>13 min) could bring better reliability of rs-fMRI connectivity analyses (Birn et al., 2013). Future studies utilizing longer scanning time for rs-fMRI data acquisition may help test the reliability of our results.

## Data Availability Statement

The datasets generated for this study are available on request to the corresponding author.

## Ethics Statement

The studies involving human participants were reviewed and approved by the Ethics Committee of the Center for Cognition and Brain Disorders at Hangzhou Normal University. The patients/participants provided their written informed consent to participate in this study.

## Author Contributions

WW and TZ contributed equally to this work as co-first author, who performed all data analysis and wrote the manuscript. XW and LL contributed the collection of MRI data and application of rTMS. QZ contributed the manuscript revision. YL contributed the conception of the study and manuscript revision. All authors read and approved the submitted version.

## Conflict of Interest

The authors declare that the research was conducted in the absence of any commercial or financial relationships that could be construed as a potential conflict of interest.

## Footnotes

1 https://www.rogue-research.com/tms/brainsight-tms

2 https://www.fil.ion.ucl.ac.uk/spm
